# The interaction between IL-17 and gut microbiota contributes to cholestatic liver disease in children

**DOI:** 10.1099/mic.0.001608

**Published:** 2025-09-16

**Authors:** Shu-Li He, Zhuo-Heng Li, Juan Li, Ying Li

**Affiliations:** 1 Kunming Children’s Hospital (Kunming Medical University Affiliated), Kunming, PR China

**Keywords:** *Bacteroides*, bile acid, children, cholestatic liver disease, gut microbiota, IL-17

## Abstract

The pathogenesis of cholestatic liver disease (CLD) is unknown, but the influence of gut microbiota and inflammation cannot be ignored. In this study, we attempted to provide theoretical insights for the diagnosis and treatment of CLD in children by analysing the association between gut microbiota, IL-17 levels and clinical characteristics. This research involved 21 children diagnosed with CLD and 11 healthy controls. Blood and faecal samples were collected from these participants. Blood samples underwent analysis for clinical indicators and IL-17 concentrations. Gut microbiota was examined through 16S rRNA gene sequencing for identification and functional prediction. A positive correlation between IL-17 levels and clinical parameters (total bile acids, alanine aminotransferase, aspartate aminotransferase and triglycerides) in children with CLD was observed. Notably, children with CLD exhibited reduced diversity and disturbances in gut microbiota, highlighted by a severe decrease of *Bacteroidota* (genus *Bacteroides*). Moreover, increased relative abundance of secondary bile acid-promoting (e.g. *Clostridium*, *Enterococcus* and *Bifidobacterium*) and deleterious (e.g. *Escherichia–Shigella* and *Streptococcus*) flora in the intestinal flora of children with CLD was positively correlated with IL-17, leading to increased inflammation and CLD aggravation. Functional predictions of gut microbiota revealed higher concentrations of l-asparagine transporter, ABC-type polar amino acid transport system and glycolysis II (from fructose 6-phosphate) functions, while the function of the Na^+^-driven multidrug efflux pump was decreased. In conclusion, children suffering from CLD exhibit significant gut microbiota disturbances, particularly a severe decrease in *Bacteroidota* (genus *Bacteroides*). Dysbiosis of the gut microbiota and elevated levels of IL-17 mutually reinforce each other, together mediating the onset and progression of CLD.

## Data Availability

The datasets generated and/or analysed in this study are available in NCBI SRA under BioProject number PRJNA1263139 and accession number SRP585454.

## Introduction

Cholestatic liver disease (CLD) arises when liver function diminishes due to cholestasis. Bile primarily contains bile acids (BAs), bilirubin and fats. The liver produces these substances, which then enter the bile ducts to facilitate lipid digestion and absorption [1]. Bile secretion relies on the functionality of various membrane transport systems present in hepatocytes and bile duct cells. Disruptions in these systems can easily lead to cholestasis [2]. It has been found that moderate BAs promote metabolic detoxification of hepatocytes [3], but their stagnation triggers intense inflammation and exacerbates hepatocyte damage [4]. Although BA derivatives for cholestasis treatment remain in clinical development, supplementation with these BA derivatives and fat-soluble vitamins (A, D, E and K) remains vital [5]. Untreated cholestasis progresses to cirrhosis and liver failure [6]. Liver transplantation is the mainstay of treatment, but re-transplantation often occurs [7]. Early diagnosis and intervention are crucial [8]. Additionally, a common feature of CLD is an elevated accumulation of pro-inflammatory cytokines [4,9]. Inflammation not only directly leads to biliary epithelial cell damage, which is the initiator of CLD, but it also modulates biliary inflammation to exacerbate the malignant progression of CLD [10]. Intestinal flora enzymes involved in bile acid metabolism regulate Th17 and Treg cells and influence intestinal immunity [11]. IL-17 is the main factor produced by Th17 cells and, as a typical pro-inflammatory cytokine, contributes to tissue infiltration and damage [12,13]. Notably, high expression of helper T cells in the liver and their major production of the inflammatory factor IL-17 is characteristic of hepatic inflammation and subsequent liver fibrosis [14,15]. Not only that, IL-17 is involved in the pathogenesis of CLD by activating specific cells [16,17]. Furthermore, in a cholestasis model of liver injury, IL-17 signalling promotes inflammatory cell production of multiple inflammatory factors such as IL-6, IL-1 and tumour necrosis factor-*α* [18]. IL-17 inhibition reduces bile ligation-induced liver injury [18]. These findings suggest that among multiple inflammatory factors, IL-17 is strongly associated with CLD. Studies targeting IL-17 may help in the diagnosis and mechanism exploration of CLD.

The liver engages directly with the intestine via the hepatic portal vein and the bile secretion system. The gut microbiota, a vital community for gastrointestinal function, comprises around 76% of the human microbiome. It synthesizes essential vitamins and amino acids critical for human health, significantly influencing cholesterol and BA metabolism [19,20]. Dysbiosis within the gut microbiota can precipitate conditions such as cirrhosis and cholangitis, among other diseases [21]. Moreover, changes in gut microbiota have been associated with the pathogenesis of parenteral nutrition-associated cholestasis [22]. Investigations into the gut microbiota of jaundiced patients indicate that developing gut microbial markers alongside serum and faecal metabolite analysis may facilitate the diagnosis of the disease [23,24]. In addition, clinical research reveals that the aetiology of liver disease varies between children and adults [25]. However, the interplay of IL-17, gut microbiota and CLD in children remains to be fully elucidated.

In this study, faecal and blood samples from 21 children diagnosed with CLD and 11 healthy controls were collected for comparative analysis. DNA samples were extracted from the faecal samples, and the DNA samples were subjected to 16S rRNA sequencing using V3 and V4 region amplicons. Investigating potential alterations in gut microbes and IL-17 levels may enhance our comprehension of CLD in paediatric populations. This understanding provides better diagnostic, preventive and therapeutic strategies for children with CLD.

## Method

### Research participants

This study utilized a descriptive and observational design. The criteria for including children were meeting the diagnostic standards for CLD and being under 18 years of age. The diagnostic criteria for CLD included an elevation of alkaline phosphatase (ALP) and/or *γ*-glutamyltransferase (*γ*-GT). Additionally, total bilirubin (TB) must be less than 85 µmol l^−1^ and direct bilirubin (DB) greater than 17 µmol l^−1^, or when TB exceeds 85 µmol l^−1^, DB must surpass 20% [26,27]. It also required alanine aminotransferase (ALT) levels to be at least 1.5–2 times the upper limit of normal and a significant increase in aspartate aminotransferase (AST) [28,29]. After excluding paediatric patients with incomplete data, only those who had not received medications or antibiotics in the previous month were selected. The final sample consisted of 31 cases, including 21 patients with CLD (CH) and 11 healthy controls (CK). The diet of all children participating in this study was based on a light diet with breastfeeding supplementation. The sample was drawn from patients presenting to Kunming Children’s Hospital from February through May 2024. Informed written consent was secured from both the children and their guardians, alongside the Ethics Committee of Kunming Children’s Hospital approval (IEC-C-008-A07-V3.0).

### Sample collection

Faecal and blood samples were collected prospectively by a trained nurse. Using a sterile disposable spatula, samples were placed in a sterile container. They were immediately frozen at −80 °C until further analysis. Relevant clinical information, such as gender, age, nationality and birth weight, was documented. A part of the blood sample underwent testing for haemoglobin, ALT, AST, TB, DB, indirect bilirubin (IB), total bile acids (TBA), *γ*-GT, ALP, albumin (ALB), prothrombin time (PT), activated partial thromboplastin time (APTT), fibrinogen (FIB), thrombin time (TT), international normalized ratio (INR), glucose (Glu) and total cholesterol (TC). The remaining blood was used to assess IL-17 levels via ELISA following the reagent manufacturer’s protocol (E-EL-H6181, Elabscience, China).

### DNA extraction, amplification and sequencing

DNA was extracted from 200 mg faecal samples using the QIAamp Fast DNA Stool Mini Kit (QIAGEN, Germany) according to the manufacturer’s instructions. In all qualified DNA samples, we localized and quantified amplicons amplified from the 16S rDNA V3 and V4 regions. Amplification was performed using universal primers 341F: 5′- CCTACGGGRSGCAGCAG-3′ and 806 R: 5′-GGACTACVVGGGGTATCTAATC-3′. After library preparation, sequencing was performed on the MiSeq platform (Illumina, Inc., CA, USA).

### Sequencing data processing

The raw data obtained from sequencing is spliced and filtered to obtain effective data. Fastp software (version 0.23.1) assisted in filtering the spliced raw tags to derive clean tags [30]. During the above data processing, fragments above 500 bp (eukaryotic) were removed. The DADA2 method was used for noise reduction [31]. Each de-emphasized sequence produced after noise reduction using DADA2 is called an amplicon sequence variant (ASV). The DADA2 method is more advantageous than the traditional OTU method in that the ASV analysis takes into account the unique sequences, which improves the accuracy and comprehensiveness of the tagged gene data analysis [32,33]. The QIIME2 package of the R software (version 4.0.3) was annotated based on the Silva (version 138.1) database for further screening of bacteria and archaea [34]. Seven archaea were identified at the kingdom level (0.40%, 7/1744). Difference analysis of the alpha diversity index between the two comparison groups was performed by the T-test, the Wilcoxon rank sum test and the Tukey test. In the beta diversity study, four metrics, weighted UniFrac distance, unweighted UniFrac distance, Jaccard distance and Bray–Curtis distance, were used to measure the coefficient of divergence between the two comparison groups. The coefficient of dissimilarity was positively correlated with species diversity. Unweighted pair-group method with arithmetic mean (UPGMA) was used to perform cluster analysis of samples [35]. PICRUSt (Phylogenetic Investigation of Communities by Reconstruction of Unobserved States) (version 1.1.4) was used for the prediction of functions.

### Statistical analysis

Statistical analyses were performed using SPSS 23.0, and *P*<0.05 was considered statistically significant. Data presentation included median and interquartile range (IQR) (25%, 75%) comparisons via the non-parametric Mann–Whitney test. Categorical variables were expressed as numbers and percentages and compared using the chi-square test. Spearman correlation analysis was performed in GraphPad Prism 9.5, with *P*<0.05 regarded as statistically significant.

## Results

### Clinical characteristics and IL-17 levels in children with CLD differ markedly from those of healthy children

**Fig. 1.**
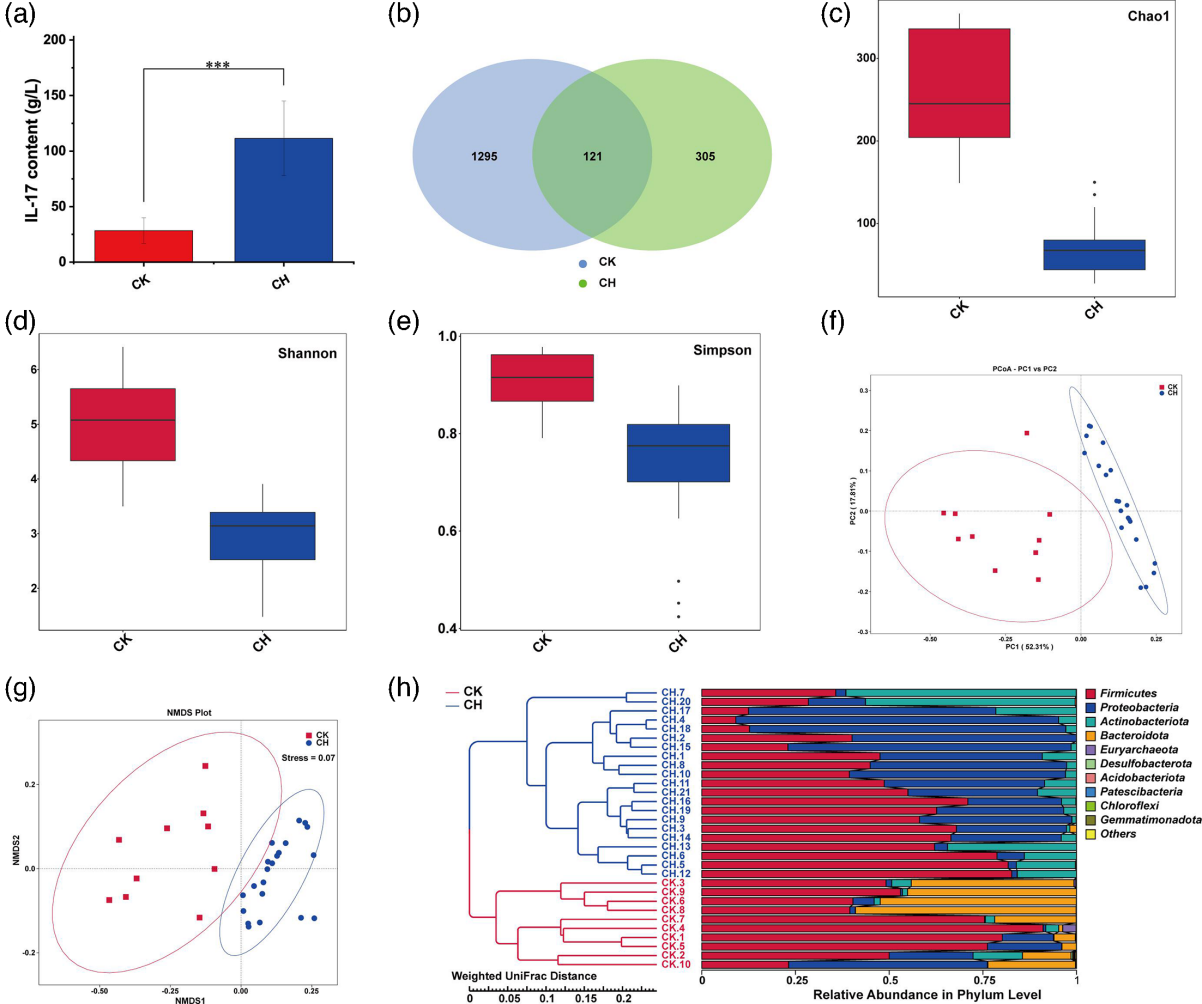
Comparative analysis of IL-17 and gut microbiota between children with CLD and healthy children. (a) Analysis of IL-17 content by ELISA. (b) Venn diagram of the number of gut microbiota. (c–e) Alpha diversity Chao 1 (**c**), Shannon (**d**) and Simpson (**e**) index analysis. (f–g) PCoA (**f**) and NMDS (**g**) beta diversity analysis. (h) UPGMA clustering tree based on weighted UniFrac distance.

Blood samples from 21 children with CLD in the CH group and 11 in the CK group were analysed. Among the 21 paediatric patients with CLD, 10 (10/21, 47.6%) were boys. The differences in sex, age, nationality and birth weight were not statistically significant (Table 1). Blood tests (Table 1) revealed that ALP (576.00 vs. 266.00 U l^−1^, *P*<0.001), TB (140.0 µmol l^−1^; IQR, 113.9, 166.8 µmol l^−1^) and DB (103.60 µmol l^−1^; IQR, 88.10, 118.50 µmol l^−1^) in the CH group were significantly higher. These results indicated that children in the CH group had significant cholestatic symptoms. Furthermore, ALT levels were significantly higher in the CH group (226.00 U l^−1^; IQR, 125.00, 624.00 U l^−1^) compared to healthy children (10.00 U l^−1^; IQR, 7.50, 11.75 U l^−1^). Likewise, AST levels were also elevated in the CH group (397.00 U l^−1^; IQR, 249.00, 1128.00 U l^−1^) compared to healthy children (26.00 U l^−1^; IQR, 24.25, 28.50 U l^−1^), indicating liver damage among the children in the CH group. Additionally, haemoglobin levels were significantly lower in the CH group (117.00 vs. 138.00 g l^−1^, *P*<0.001), along with ALB levels (37.70 vs. 44.40 g l^−1^, *P*<0.001) relative to the CK group. In contrast, children with CLD exhibited significantly elevated levels of TBA (241.80 vs. 3.05 µmol l^−1^, *P*<0.001), *γ*-GT (187.00 vs. 12.00 U l^−1^, *P*<0.001), PT (14.90 vs. 13.25, *P*<0.001), APTT (44.50 vs. 37.45, *P*=0.008), TT (19.10 vs. 17.50, *P*=0.003), INR (1.16 vs. 1.05, *P*=0.001) and triglycerides (1.69 vs. 1.00 mmol l^−1^, *P*=0.035). There were no significant differences in FIB, Glu or TC levels between the groups. Lastly, measuring IL-17 levels revealed that the CH group had notably higher concentrations of IL-17 than the CK group (Fig. 1a).

**Table 1.** Clinical characteristics of children in the CH and CK groups

Characteristic	CH	CK	*P*
No. (%) of children by sex			0.602
Boys	10 (47.62)	5 (45.45)	
Girls	11 (52.38)	6 (54.55)	
Median (IQR) age (years)	2.99 (2.50, 3.40)	3.18 (3.00, 3.20)	0.327
No. (%) of children by nationality			0.526
Han	14 (66.67)	8 (72.73)	
National minority	7 (33.33)	3 (27.27)	
Birth weight (Kg)	2.75 (2.50, 3.40)	3.05 (3.00, 3.20)	0.327
Haemoglobin (g l^−1^)	117.00 (110.00, 124.00)	138.00 (135.25, 141.75)	＜0.001
ALT (U l^−1^)	226.00 (125.00, 624.00)	10.00 (7.50, 11.75)	＜0.001
AST (U l^−1^)	397.00 (249.00, 1128.00)	26.00 (24.25, 28.50)	＜0.001
TB (μmol l^−1^)	140.00 (113.90, 166.80)	13.00 (10.25, 18.33)	＜0.001
DB (μmol l^−1^)	103.60 (88.10, 118.50)	3.25 (2.90, 4.33)	＜0.001
IB (μmol l^−1^)	36.70 (26.20, 44.90)	10.30 (8.50, 14.75)	＜0.001
TBA (μmol l^−1^)	241.80 (125.90, 381.90)	3.05 (1.93, 4.18)	＜0.001
*γ*-GT (U l^−1^)	187.00 (112.00, 256.00)	12.00 (11.00, 13.00)	＜0.001
ALP (U l^−1^)	576.00 (438.00, 726.00)	266.00 (221.00, 307.50)	＜0.001
ALB (g l^−1^)	37.70 (35.40, 39.20)	44.40 (42.98, 45.78)	＜0.001
PT	14.90 (14.00, 17.30)	13.25 (12.95, 13.7)	＜0.001
APTT	44.50 (40.80, 46.70)	37.45 (36.28, 41.08)	0.008
FIB	1.92 (1.43, 2.78)	2.39 (2.25, 2.67)	0.186
TT	19.10 (18.10, 20.00)	17.50 (17.15, 17.58)	0.003
INR	1.16 (1.10, 1.48)	1.05 (1.01, 1.08)	0.001
Glu (mmol l^−1^)	4.30 (3.50, 4.80)	4.45 (3.88, 4.50)	0.917
TC (mmol l^−1^)	4.36 (3.75, 5.40)	4.92 (4.79, 5.25)	0.147
Triglyceride (mmol l^−1^)	1.69 (1.04, 2.15)	1.00 (0.77, 1.36)	0.035

Data were compared using the nonparametric Mann–Whitney test. The chi-square test was used to compare the distribution of dichotomous variables.

### Notable variations in gut microbiota between children with CLD and healthy controls

We obtained 2,858,838 high-quality reads from 31 faecal samples with 92,221±19,066 reads per sample (mean±sd). The proportion of bases with Q20 (indicating a sequencing error rate below 1%) in the effective tags was 98%. A total of 1,721 ASVs were identified in both groups, with 1,416 ASVs in the CK group significantly higher than 426 ASVs in the CH group, which included a shared 121 ASVs (Fig. 1b). The percentage of identification: phylum (99.31%), class (99.14%), order (98.11%), family (96.73%), genus (85.26%) and species (16.06%). Emphatically, 16S rRNA genes are highly conserved among bacteria in gut flora analyses, especially with minimal differences among closely related species, resulting in both species-level identification rates and accuracies that are typically low [36]. Based on the ASVs, we compared the alpha and beta diversity in the gut microbiota of the two groups. Analyses of alpha diversity indices, namely Chao 1, Shannon and Simpson, revealed that children with CLD showed notably lower species richness (Fig. 1c) and diversity (Fig. 1d) compared to healthy children. Furthermore, they exhibited greater homogeneity (Fig. 1e). Principal coordinate analysis (PCoA) and non-metric multidimensional scaling (NMDS) were employed for comprehensive beta diversity evaluation, which displayed distinct clustering in gut microbiota composition. The primary axis of the 2D PCoA plot (PC1) accounted for 52.31% of the variance, clearly separating the CH and CK groups, signifying significant differences in gut microbiota composition between the two groups (Fig. 1f). The NMDS results also corroborated significant intergroup variability, despite some overlap (Fig. 1g). Additionally, the UPGMA clustering tree based on weighted UniFrac distance illustrated analogous findings – high similarity within groups and marked differences between them (Fig. 1h). Meanwhile, both groups demonstrated a substantial prevalence of *Firmicutes* at the phylum level. In contrast, *Proteobacteria* represented a larger proportion in the CH group, while *Bacteroidota* was more predominant in the CK group.

### The gut microbiota of children with CLD exhibits dysbiosis, showing significant differences compared to healthy controls

**Fig. 2.**
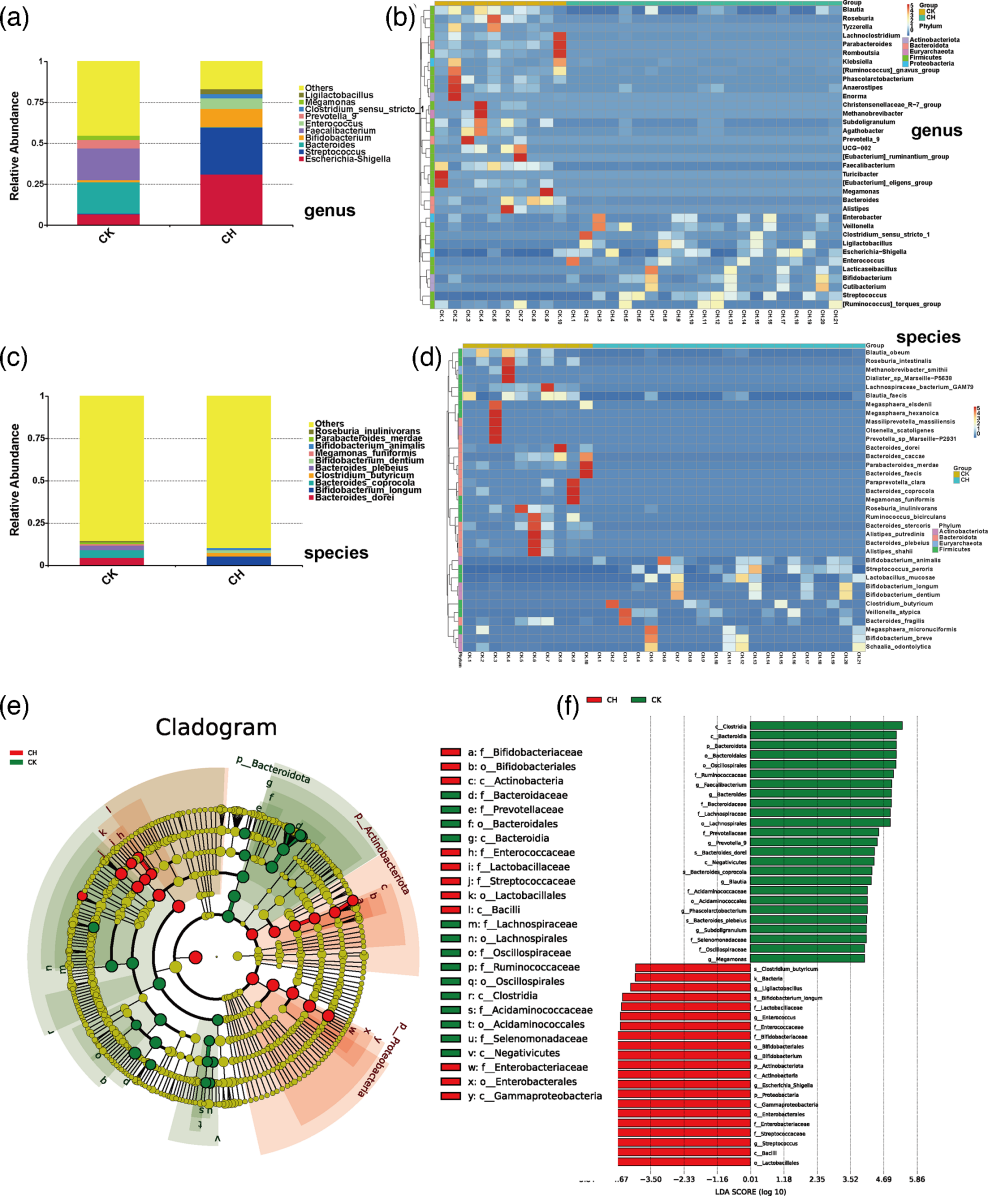
Analysis of the gut microbiota at the genus and species level and LEfSe analysis. (a–b) Histogram (**a**) and heat map (**b**) at the genus level of the gut microbiota. (c–d) Histogram (**c**) and heat map (**d**) at the species level of the gut microbiota. (e–f) Evolutionary branching diagram (**e**) and histogram of the distribution of the linear discriminant analysis values (**f**) of the LEfSe analysis. The phylum, class, order, family, genus and species they belong to are listed in the legend.

As shown in the histogram (Fig. 2a) and heat map (Fig. 2b) at the genus level. The heat map showed a cross shape, which indicated a better differentiation between the groups. The dominant bacteria in the CH group were *Escherichia–Shigella* and *Streptococcus*, while the dominant bacteria in the CK group were *Bacteroides* and *Faecalibacterium*. More specifically, the intestinal flora of children with CLD showed a significant increase in the relative abundance of *Escherichia–Shigella*, *Streptococcus* and *Bifidobacterium* and the addition of the dominant groups *Enterococcus*, *Ligilactobacillus* and *Clostridium sensu stricto 1*, as compared to the intestinal flora of healthy children. In addition, the CH group showed a significant decrease in the relative abundance of *Megamonas*, *Prevotella 9*, *Faecalibacterium* and *Bacteroides*, which lost their status as the dominant flora. The histogram (Fig. 2c) and heat map (Fig. 2d) at the species level showed significantly diverging gut microbiota compositions with no overlapping dominant species. Specifically, *Bacteroides_coprocola* and *Bacteroides_dorei* were more abundant in the CK group, while *Bifidobacterium_longum* was predominant in the CH group. Analysis of gut microbiota at the phylum, class and family levels was in Material S1, available in the online Supplementary Material.

Linear discriminant analysis effect size (LEfSe) (Fig. 2e, f) identified nine bacterial taxa enriched in the CH group, spanning various taxonomic levels, including the phylum level of *Proteobacteria*, the class level of *Bacilli* and *Gammaproteobacteria*, the order level of *Lactobacillales* and *Enterobicterales*, the family level of *Streptococcus* and *Enterobacteriaceae* and the genus level of *Escherichia_Shigella* and *Streptococcus*. Conversely, 11 bacterial taxa, notably including *Bacteroidota* at the phylum level; *Clostridia* and *Bacteroidia* at the class level; *Bacteroidales*, *Oscillospirales* and *Lachnospirales* at the order level; *Ruminococcaceae*, *Bacteroidaceae* and *Lachnospiraceae* at the family level; and *Faecalibacterium* and *Bacteroides* at the genus level, showed substantial reductions in the CH group.

### Altered patterns of gut microbial symbiosis and function in children with CLD

**Fig. 3.**
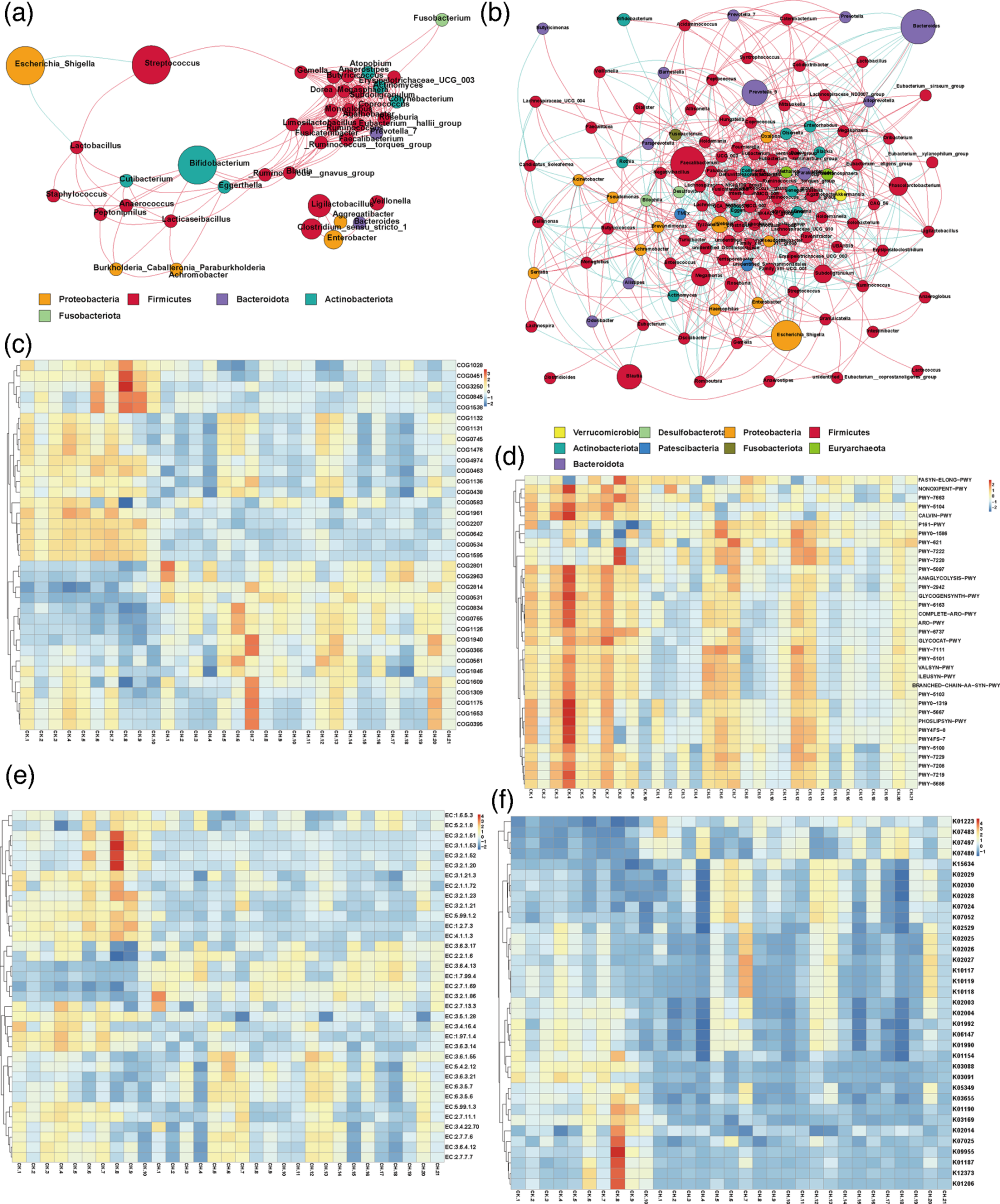
Network analysis and functional prediction of gut microbiota. (a–b) Network analysis of gut microbiota in children with CLD (**a**) and healthy children (**b**). (c–f) Functional prediction analysis of gut microbiota in the direction of COG (**c**), pathways (**d**), enzymes (**e**) and KO (**f**).

The gut microbiota networks of children in the CH group, compared to healthy controls, exhibited distinct symbiotic patterns (Fig. 3a, b). The simpler bacterial community structure in the CLD group indicates that CLD alters gut microbiota composition and disrupts balance. We utilized the software PICRUSt to forecast gut microbiota functions in both groups from four perspectives: Clusters of Orthologous Groups (COG), pathways, enzymes and KEGG Orthology (KO). In terms of COG, the gut microbiota functions in children with CLD emphasized predicted arabinose efflux permease (COG2814), ABC-type amino acid transport system (COG0765), l-asparagine transporter and related permeases (COG1113) and DNA-binding transcriptional regulator (COG1609), contrasting with healthy children. Conversely, the proportions of DNA-directed RNA polymerase specialized sigma subunit (COG1595) and Na^+^-driven multidrug efflux pump (COG0534) were lower (Fig. 3c). From the pathway perspective, children with CLD displayed higher levels of pathways such as inosine-5′-phosphate biosynthesis III (PWY-7234), superpathway of l-alanine biosynthesis (PWY0-1061), the peptidoglycan maturation (meso-diaminopimelate containing) (PWY0-1586), acetylene degradation (P161-PWY) and glycolysis II (from fructose 6- phosphate) (PWY-5484) (Fig. 3d). In terms of enzymes, the control group (CK) revealed higher proportions of protein-N(pi)-phosphohistidine--sugar phosphotransferase (EC:2.7.1.69), DNA-directed DNA polymerase (EC:2.7.7.7), 6-phospho-beta-glucosidase (EC:3.2.1.86) and DNA helicase (EC:3.6.4.12), while the ratios of 2-oxoglutarate synthase (EC:1.2.7.3) and alpha-l-fucosidase (EC. 3.2.1.51) were lower than those in the CLD group (Fig. 3e). Kyoto Encyclopedia of Genes and Genomes (KEGG) functional prediction analysis indicated that the CK group experienced a substantial decrease in RNA polymerase sigma-70 factor (K03088), while proportions of sucrose−6-phosphatase (K07024), transposase (K07483) and 6-phospho-beta-glucosidase (K01223) increased (Fig. 3f).

### Gut microbiota, clinical characteristics and IL-17 correlation in children with CLD

**Fig. 4.**
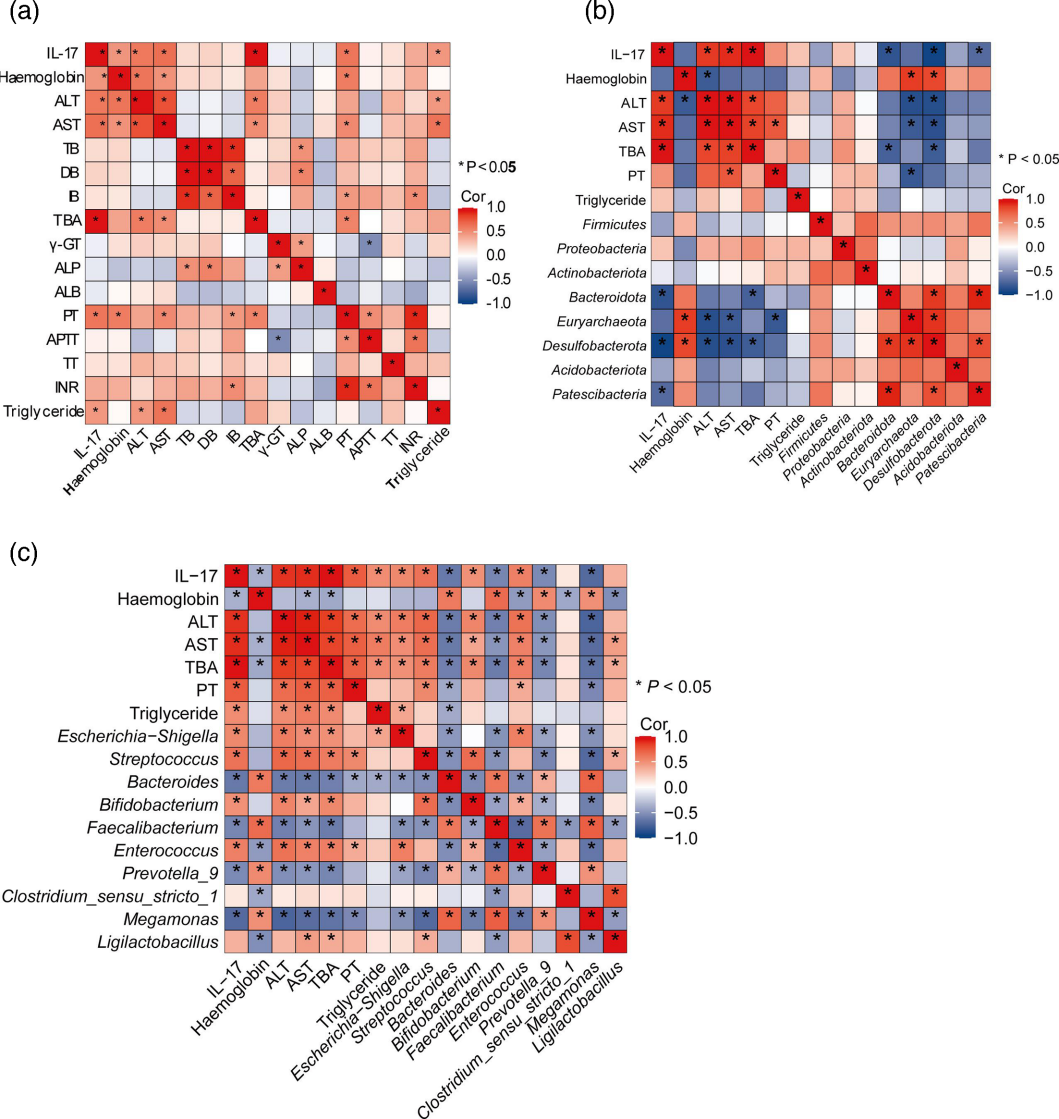
Correlation analysis of gut microbiota, clinical characteristics and IL-17. (a) Correlation analysis of IL-17 and clinical characteristics. (b–c) Correlation analysis of gut microbiota at phylum (b) or genus (c) level, IL-17 and clinical characteristics. P<0.05 was considered statistically significant and marked as *.

Spearman’s non-parametric correlation analysis of blood parameters and IL-17 revealed positive correlations in the CLD group between IL-17 content and levels of TBA (*r*=0.95, *P*<0.05), haemoglobin (*r*=0.47, *P*<0.05), ALT (*r*=0.60, *P*<0.05), AST (*r*=0.64, *P*<0.05), PT (*r*=0.61, *P*<0.05) and triglyceride (*r*=0.45, *P*<0.05) (Fig. 4a). Further analysis of the correlation matrix indicated that IL-17 negatively correlated with *Bacteroidota*, *Desulfobacterota* and *Patescibacteria* (Fig. 4b). Moreover, *Bacteroidota*, *Euryarchaeota* and *Desulfobacterota*, linked to CLD, showed a negative relationship with the progression of CLD in paediatric patients. At the genus level, IL-17 content was significantly positively correlated with *Escherichia–Shigella*, *Streptococcus*, *Bifidobacterium* and *Enterococcus*, while it was significantly negatively correlated with *Bacteroides*, *Faecalibacterium*, *Prevotella_9* and *Megamonas* (Fig. 4c).

## Discussion

Here, we investigated the interplay among clinical characteristics, gut microbiota and IL-17 in children with CLD. The expression of IL-17 in children with CLD was approximately four times higher than that in healthy controls. Previous research also identified overexpression of IL-17 in the serum of primary biliary cirrhosis patients [37]. Moreover, Spearman correlation analysis revealed a positive association between IL-17 levels and clinical parameters such as TBA, ALT, AST and triglycerides in children with CLD, consistent with earlier studies [37,39]. Our investigation revealed a significant reduction of *Bacteroidota* (genus *Bacteroides*) in children with CLD, indicating its potential as a therapeutic target or a microbiota marker for CLD. Notably, prior studies have illustrated the relevance of *Bacteroidota* in various conditions [40], including cholangiocarcinoma [41], ulcerative colitis [42], infant neurodevelopment [43] and Alzheimer’s disease [44]. Furthermore, the reduced *Bacteroidota*-to-*Firmicutes* ratio correlates with chronic lymphocytic leukaemia [45] and acromegaly [46], alongside an observed decline in immune function [47]. The significantly lowered B/F ratio in children with CLD potentially predisposes them to the onset of multiple diseases. Moreover, similar to findings in infants with cholestasis [48], our study underscores that CLD results in gut microbiota dysbiosis, altering microbial community structure and diminishing diversity within this vital ecosystem. Besides, LEfSe analysis revealed an intrinsic link among IL-17, gut microbiota and CLD (more discussion was in Material S2).

Correlation analysis shows that *Bacteroidota* and *Desulfobacterota* exhibit a negative correlation with IL-17 and the clinical characteristics of children suffering from CLD. Inflammation, beyond being a pathological feature of CLD, contributes to further disease progression, ultimately leading to cirrhosis or hepatocellular carcinoma [49]. Previous studies have also found that *Bacteroidota* can play an important anti-inflammatory role in colitis [42]. *Desulfobacterota*, although rarely reported with inflammation, acts as a bile salt hydrolase (BSH)-containing bacterium with BA decoupling ability in the process of hepatic injury due to cholestasis, promoting the conversion of primary BAs to secondary BAs [50]. Intestinal bacteria deconjugate primary BAs to free BAs via BSH and then convert free BAs to secondary BAs via the steps of 7*α*-dehydroxylation, oxidation and isomerization [51]. Previous studies have found that the gram-positive bacteria *Clostridium*, *Enterococcus* and *Bifidobacterium* contain BSH [52], and *Clostridium* to be a 7*α*-dehydroxylating bacterium with 7*α*-dehydroxylating activity [53]. In our study, we observed an increase in the relative abundance of BSH-containing bacteria, *Clostridium sensu stricto 1*, *Enterococcus* and *Bifidobacterium*, which causes accelerated conversion and accumulation of secondary BAs. Excessive accumulation of secondary BAs and inflammation can lead to increased cholestasis and liver damage. Additionally, we observed a significant increase in the relative abundance of *Escherichia–Shigella* and *Streptococcus* in the intestinal flora of children with CLD compared to that of healthy children, and a significant positive correlation with IL-17 levels. Increased relative abundance of *Clostridium*, *Enterococcus* and *Escherichia–Shigella* was observed in patients with gallstones and in paediatric patients with biliary atresia, which matches our findings [51,54]. Moreover, the relative abundance of *Faecalibacterium* and *Bacteroides* was significantly reduced in the CH group and was significantly negatively correlated with IL-17. Reduced abundance of *Bacteroides* and *Faecalibacterium* was associated with increased intestinal inflammation and reduced liver function [55,56]. In addition, elevated *Escherichia–Shigella* relative abundance was associated with elevated lipopolysaccharide levels, promoting further inflammation [57]. Previous studies have shown that Th17, the main IL-17-producing cell, is affected by BAs, and that secondary bile acid metabolism is regulated by intestinal flora [11,58]. Furthermore, previous studies have observed a significant decrease in *Bacteroides* and a significant increase in *Streptococcus* in children with cholangitis and colitis [59]. The abundance ratio of *Streptococcus/B*acteroides may be a diagnostic indicator of neonatal biliary atresia [60]. These findings indicated that changes in the intestinal flora of children with CLD were an important factor and outcome in the aggravation of CLD. These findings suggest that an increase in the relative abundance of secondary bile acid production-promoting bacteria, such as *Clostridium*, *Enterococcus* and *Bifidobacterium*, promotes biliary stasis and the malignant progression of CLD. Inflammation-associated bacteria, such as *Escherichia–Shigella*, *Bacteroides* and *Faecalibacterium*, whose altered relative abundance regulates IL-17 production and leads to further exacerbation and worsening of inflammation. In brief, changes in the intestinal flora of children with CLD are an important factor and outcome of the exacerbation of CLD. Moreover, the results of functional prediction indicated that gut microbiota function in children with CLD mainly relates to the l-asparagine transporter and related permeases. Earlier studies have associated l-asparaginase-induced hepatotoxicity with cholestasis and accelerated liver disease [61,62]. Furthermore, studies have found that malnutrition leading to amino acid deficiencies [63], glucose metabolism [64] and Na^+^-driven multidrug efflux pump [65] was associated with cholestasis and liver function, which aligns with our study. However, it remains unclear whether these variables act as disease contributors or consequences. Furthermore, these functional enrichments are predictive, and further experimental validation is necessary.

In this study, we analysed the blood and faeces of children with CLD or health and predicted the function of gut microbiota to investigate the relationship between clinical characteristics, IL-17 and gut microbiota. Characteristically*,* we observed a significant reduction of *Bacteroidota* (genus *Bacteroides*) in the gut of children with CLD. Moreover, increased relative abundance of secondary bile acid-promoting (e.g. *Clostridium*, *Enterococcus* and *Bifidobacterium*) and deleterious (e.g. *Escherichia–Shigella* and *Streptococcus*) flora in the intestinal flora of children with CLD was positively correlated with IL-17, leading to increased inflammation and CLD aggravation. The limited sample size constrains the applicability of these results, thus necessitating future studies with larger cohorts to validate our conclusions. Moreover, this study found elevated TBA levels in CLD, but did not analyse specific bile acid species. Further analytical studies (e.g. LC-MS) could provide a more detailed understanding of BA composition and its relationship to microbial taxa and inflammation. In addition, determining whether IL-17 is produced only by Th17 cells during this process will also help to better explain and enrich the results of this study. Furthermore, IL-17 has been found to enhance the integrity and function of the intestinal mucosal barrier and promote the balance of the intestinal microbiota by inhibiting inflammatory/immune cell infiltration and reducing intestinal permeability [66], which warrants discussion in future studies. Nonetheless, our study holds substantial significance for the diagnosis, treatment and prognosis of CLD in paediatric patients. Transplantation of *Bacteroidota* (genus *Bacteroides*) along with the inhibition of IL-17 expression may serve as effective therapeutic strategies for childhood CLD and prevent disease progression.

## Conclusion

In this study, we observed the gut microbiota in CLD. Key findings included a marked reduction of *Bacteroidota* (genus *Bacteroides*) and the increased relative abundance of secondary bile acid-promoting flora and deleterious flora in the intestinal flora of children with CLD was positively correlated with IL-17, leading to increased inflammation and CLD aggravation.

## Supplementary material

10.1099/mic.0.001608 Uncited Supplementary Material 1.

## Abbreviations

ALB: albumin
ALP: alkaline phosphatase
ALT: alanine aminotransferase
APTT: activated partial thromboplastin time
AST: aspartate aminotransferase
ASV: amplicon sequence variant
BAs: bile acids
BSH: bile salt hydrolase
CH: patients with CLD
CK: healthy controls
CLD: cholestatic liver disease
DB: direct bilirubin
FIB: fibrinogen
Glu: glucose
IB: indirect bilirubin
INR: international normalized ratio
IQR: interquartile range
LEfSe: linear discriminant analysis effect size
NMDS: non-metric multidimensional scaling
PCoA: principal coordinate analysis
PICRUSt: Phylogenetic Investigation of Communities by Reconstruction of Unobserved States
PT: prothrombin time
TB: total bilirubin
TBA: total bile acids
TC: total cholesterol
TT: thrombin time
UPGMA: unweighted pair-group method with arithmetic mean
*γ*-GT: *γ*-glutamyltransferase

## References

[1] Boyer JL (2013). Bile formation and secretion. Compr Physiol.

[2] Li MK, Crawford JM (2004). The pathology of cholestasis. Semin Liver Dis.

[3] Wang H, Chen J, Hollister K, Sowers LC, Forman BM (1999). Endogenous bile acids are ligands for the nuclear receptor FXR/BAR. Mol Cell.

[4] Gujral JS, Farhood A, Bajt ML, Jaeschke H (2003). Neutrophils aggravate acute liver injury during obstructive cholestasis in bile duct-ligated mice. Hepatology.

[5] Yu L, Liu Y, Wang S, Zhang Q, Zhao J (2023). Cholestasis: exploring the triangular relationship of gut microbiota-bile acid-cholestasis and the potential probiotic strategies. Gut Microbes.

[6] Afonso MB, Rodrigues PM, Simão AL, Ofengeim D, Carvalho T (2016). Activation of necroptosis in human and experimental cholestasis. Cell Death Dis.

[7] Squires JE, McKiernan P (2018). Molecular mechanisms in pediatric cholestasis. Gastroenterol Clin North Am.

[8] Serinet M-O, Wildhaber BE, Broué P, Lachaux A, Sarles J (2009). Impact of age at Kasai operation on its results in late childhood and adolescence: a rational basis for biliary atresia screening. Pediatrics.

[9] Gehring S, Dickson EM, San Martin ME, van Rooijen N, Papa EF (2006). Kupffer cells abrogate cholestatic liver injury in mice. Gastroenterology.

[10] Chen J, Zhang S (2023). The role of inflammation in cholestatic liver injury. J Inflamm Res.

[11] Zhang Y, Gao X, Gao S, Liu Y, Wang W (2023). Effect of gut flora mediated-bile acid metabolism on intestinal immune microenvironment. Immunology.

[12] Voo KS, Wang Y-H, Santori FR, Boggiano C, Wang Y-H (2009). Identification of IL-17-producing FOXP3 ^+^ regulatory T cells in humans. Proc Natl Acad Sci USA.

[13] Korn T, Bettelli E, Oukka M, Kuchroo VK (2009). IL-17 and Th17 Cells. Annu Rev Immunol.

[14] Ge J, Wang K, Meng Q-H, Qi Z-X, Meng F-L (2010). Implication of Th17 and Th1 cells in patients with chronic active hepatitis B. J Clin Immunol.

[15] Lemmers A, Moreno C, Gustot T, Maréchal R, Degré D (2009). The interleukin-17 pathway is involved in human alcoholic liver disease. Hepatology.

[16] Hara M, Kono H, Furuya S, Hirayama K, Tsuchiya M (2013). Interleukin-17A plays a pivotal role in cholestatic liver fibrosis in mice. J Surg Res.

[17] Beringer A, Miossec P (2018). IL-17 and IL-17-producing cells and liver diseases, with focus on autoimmune liver diseases. Autoimmun Rev.

[18] Meng F, Wang K, Aoyama T, Grivennikov SI, Paik Y (2012). Interleukin-17 signaling in inflammatory, Kupffer cells, and hepatic stellate cells exacerbates liver fibrosis in mice. Gastroenterology.

[19] Gérard P (2013). Metabolism of cholesterol and bile acids by the gut microbiota. Pathogens.

[20] Li M, Liu S, Wang M, Hu H, Yin J (2020). Gut microbiota dysbiosis associated with bile acid metabolism in neonatal cholestasis disease. Sci Rep.

[21] The Human Microbiome Project Consortium (2012). Structure, function and diversity of the healthy human microbiome. Nature.

[22] Mokha JS, Davidovics ZH, Maas K, Caimano MJ, Matson A (2019). Fecal microbiomes in premature infants with and without parenteral nutrition-associated cholestasis. J Pediatr Gastroenterol Nutr.

[23] Li M, Liu S, Wang M, Hu H, Yin J (2020). Gut microbiota dysbiosis associated with bile acid metabolism in neonatal cholestasis disease. Sci Rep.

[24] Zhou S, Wang Z, He F, Qiu H, Wang Y (2019). Association of serum bilirubin in newborns affected by jaundice with gut microbiota dysbiosis. J Nutr Biochem.

[25] Leung DH, Yimlamai D (2017). The intestinal microbiome and paediatric liver disease. Lancet Gastroenterol Hepatol.

[26] Van Winckel M, Vande Velde S, De Bruyne R, Van Biervliet S (2011). Clinical practice: vegetarian infant and child nutrition. Eur J Pediatr.

[27] Chen Dong ZH (2018). Diagnosis and differential diagnosis of cholestatic liver disease in infants. Chinese Clin J Pract Pediatr.

[28] Heathcote EJ (2007). Diagnosis and management of cholestatic liver disease. Clin Gastroenterol Hepatol.

[29] Catzola A, Vajro P (2017). Management options for cholestatic liver disease in children. Expert Rev Gastroenterol Hepatol.

[30] Bokulich NA, Subramanian S, Faith JJ, Gevers D, Gordon JI (2013). Quality-filtering vastly improves diversity estimates from Illumina amplicon sequencing. Nat Methods.

[31] Callahan BJ, McMurdie PJ, Rosen MJ, Han AW, Johnson AJA (2016). DADA2: high-resolution sample inference from Illumina amplicon data. Nat Methods.

[32] Amir A, McDonald D, Navas-Molina JA, Kopylova E, Morton JT (2017). Deblur rapidly resolves single-nucleotide community sequence patterns. mSystems.

[33] Callahan BJ, Wong J, Heiner C, Oh S, Theriot CM (2019). High-throughput amplicon sequencing of the full-length 16S rRNA gene with single-nucleotide resolution. Nucleic Acids Res.

[34] Edgar RC, Haas BJ, Clemente JC, Quince C, Knight R (2011). UCHIME improves sensitivity and speed of chimera detection. Bioinformatics.

[35] Backeljau T, De Bruyn L, De Wolf H, Jordaens K, Van Dongen S (1996). Multiple UPGMA and neighbor-joining trees and the performance of some computer packages. Mol Biol Evol.

[36] Fox JD, Sims A, Ross M, Bettag J, Wilder A (2024). Bioinformatic methodologies in assessing gut microbiota. Microbiol Res.

[37] Sun Q, Wang Q, Feng N, Meng Y, Li B (2019). The expression and clinical significance of serum IL-17 in patients with primary biliary cirrhosis. Ann Transl Med.

[38] ru LIC, Li WU, li ZY, sha ZS, xia ZH (2021). Microecological preparations alleviate cholestatic liver disease by down regulating IL 17 in γδt cells of mouse liver. Chinese J Microecol.

[39] Cebi M, Yilmaz Y (2024). Immune system dysregulation in the pathogenesis of non-alcoholic steatohepatitis: unveiling the critical role of T and B lymphocytes. Front Immunol.

[40] Chen W, Wang D, Deng X, Zhang H, Dong D (2024). Bile acid profiling as an effective biomarker for staging in pediatric inflammatory bowel disease. Gut Microbes.

[41] Zhang N, Zhu W, Zhang S, Liu T, Gong L (2023). A novel *Bifidobacterium/Klebsiella* ratio in characterization analysis of the gut and bile microbiota of CCA patients. Microb Ecol.

[42] Ishikawa D, Zhang X, Nomura K, Shibuya T, Hojo M (2024). Anti-inflammatory effects of *Bacteroidota* strains derived from outstanding donors of fecal microbiota transplantation for the treatment of ulcerative colitis. Inflamm Bowel Dis.

[43] Oliphant K, Ali M, D’Souza M, Hughes PD, Sulakhe D (2021). *Bacteroidota* and *Lachnospiraceae* integration into the gut microbiome at key time points in early life are linked to infant neurodevelopment. Gut Microbes.

[44] Wasén C, Beauchamp LC, Vincentini J, Li S, LeServe DS (2024). Bacteroidota inhibit microglia clearance of amyloid-beta and promote plaque deposition in Alzheimer’s disease mouse models. Nat Commun.

[45] Paziewska M, Szelest M, Kiełbus M, Masternak M, Zaleska J (2024). Increased abundance of *Firmicutes* and depletion of *Bacteroidota* predicts poor outcome in chronic lymphocytic leukemia. Oncol Lett.

[46] Babayeva A, Ozkul C, Coskun M, Uzun A, Yalcin MM (2024). Alteration in gut microbial characteristics of patients with acromegaly. Endocrine.

[47] Rajput M, Momin T, Singh A, Banerjee S, Villasenor A (2023). Determining the association between gut microbiota and its metabolites with higher intestinal Immunoglobulin A response. Vet Anim Sci.

[48] Jin M, Cui J, Ning H, Wang M, Liu W (2023). Alterations in gut microbiota and metabolite profiles in patients with infantile cholestasis. BMC Microbiol.

[49] Wallace K, Burt AD, Wright MC (2008). Liver fibrosis. Biochem J.

[50] Martinez-Gili L, Pechlivanis A, McDonald JAK, Begum S, Badrock J (2023). Bacterial and metabolic phenotypes associated with inadequate response to ursodeoxycholic acid treatment in primary biliary cholangitis. Gut Microbes.

[51] Guo X, Okpara ES, Hu W, Yan C, Wang Y (2022). Interactive relationships between intestinal flora and bile acids. IJMS.

[52] Smith BJ, Piceno Y, Zydek M, Zhang B, Syriani LA (2022). Strain-resolved analysis in a randomized trial of antibiotic pretreatment and maintenance dose delivery mode with fecal microbiota transplant for ulcerative colitis. Sci Rep.

[53] Wells JE, Williams KB, Whitehead TR, Heuman DM, Hylemon PB (2003). Development and application of a polymerase chain reaction assay for the detection and enumeration of bile acid 7alpha-dehydroxylating bacteria in human feces. Clin Chim Acta.

[54] Wang Q, Hao C, Yao W, Zhu D, Lu H (2020). Intestinal flora imbalance affects bile acid metabolism and is associated with gallstone formation. BMC Gastroenterol.

[55] Wexler HM (2007). Bacteroides: the good, the bad, and the nitty-gritty. Clin Microbiol Rev.

[56] Miquel S, Martín R, Rossi O, Bermúdez-Humarán LG, Chatel JM (2013). *Faecalibacterium prausnitzii* and human intestinal health. Curr Opin Microbiol.

[57] Kayagaki N, Wong MT, Stowe IB, Ramani SR, Gonzalez LC (2013). Noncanonical inflammasome activation by intracellular LPS independent of TLR4. Science.

[58] Paik D, Yao L, Zhang Y, Bae S, D’Agostino GD (2022). Human gut bacteria produce ΤΗ17-modulating bile acid metabolites. *Nature*.

[59] Del Chierico F, Cardile S, Baldelli V, Alterio T, Reddel S (2024). Characterization of the gut microbiota and mycobiota in Italian pediatric patients with primary sclerosing cholangitis and ulcerative colitis. Inflamm Bowel Dis.

[60] sun D, Xie C, Zhao Y, Liao J, Li S (2024). The gut microbiota-bile acid axis in cholestatic liver disease. Mol Med.

[61] Kamal N, Koh C, Samala N, Fontana RJ, Stolz A (2019). Asparaginase-induced hepatotoxicity: rapid development of cholestasis and hepatic steatosis. Hepatol Int.

[62] Sunati Sahoo JH (2003). Histopathological features of L-asparaginase-induced liver disease. Semin Liver Dis.

[63] Liu G, Wang X, Fan X, Luo X (2022). Metabolomics profiles in acute-on-chronic liver failure: unveiling pathogenesis and predicting progression. Front Pharmacol.

[64] Hoffmann U, Kroemer HK (2004). the abc transporters mdr1 and mrp2: multiple functions in disposition of xenobiotics and drug resistance. Drug Metab Rev.

[65] Gartung C, Ananthanarayanan M, Rahman MA, Schuele S, Nundy S (1996). Down-regulation of expression and function of the rat liver Na+/bile acid cotransporter in extrahepatic cholestasis. Gastroenterology.

[66] He S, Cui S, Song W, Jiang Y, Chen H (2022). Interleukin-17 weakens the NAFLD/NASH process by facilitating intestinal barrier restoration depending on the gut microbiota. mBio.

