# Supplementary material for: The interaction between IL-17 and gut microbiota contributes to cholestatic liver disease in children
Source: Microbiology (Reading). 2025 Sep 16;171(9):001608. doi: 10.1099/mic.0.001608 (PMC12453119; doi:10.1099/mic.0.001608)
Supplement: Uncited Supplementary Material 1. [file mic-171-01608-s001.pdf]

S1

3 Results

3.3 The gut microbiota of children with CLD exhibits dysbiosis, showing significant differences compared to healthy controls

At the phylum level (Fig. S1A and B), the microbiota in the control (CK) group primarily consisted of four dominant bacterial phyla: *Firmicutes*, *Bacteroidota*, *Proteobacteria*, and *Actinobacteriota*. Conversely, the CLD group (CH) displayed three main phyla: *Firmicutes*, *Proteobacteria*, and *Actinobacteriota*, indicating a notable absence of *Bacteroidota*. This absence resulted in a marked decrease in the B/F ratio. At the class level (Fig. S1C and D), the CK group presented greater microbiota diversity, primarily comprising *Clostridia*, *Bacteroidia*, *Negativicutes*, *Gammaproteobacteria*, *Bacilli*, *Coriobacteriia*, *Actinobacteria* and *Methanobacteria*. In contrast, the CH group exhibited less diversity, mainly represented by *Gammaproteobacteria*, *Bacilli*, *Actinobacteria*, *Clostridia*, and *Negativicutes*. While some classes from the CK group were also present in the CH group, their proportions significantly differed. Notably, the relative abundance of *Clostridia* and *Negativicutes* was lower in the CH group, whereas *Gammaproteobacteria*, *Bacilli,* and *Actinobacteria* were considerably higher, with the absence of *Bacteroidia*. At the family level (Fig. S1E and F), key families in the CK group included *Bacteroidaceae*, *Ruminococcaceae,* and *Lachnospiraceae*, while the CH group was dominated by *Enterobacteriaceae*, *Streptococcaceae,* and *Bifidobacteriaceae*.

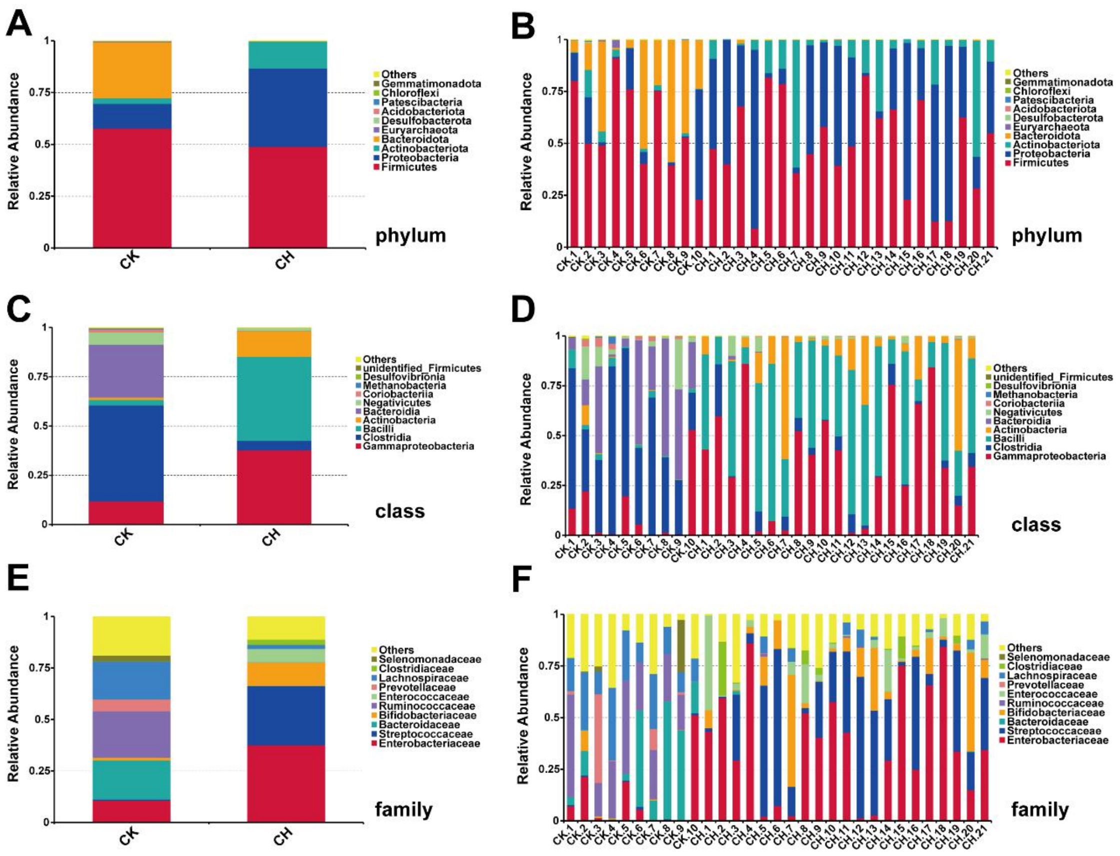

Figure S1 Analysis of gut microbiota at phylum, class, and family level. **A-B**: Analysis of gut microbiota at the phylum level. **C-D**: Analysis of gut microbiota at class level. **E-F**: Analysis of gut microbiota at the family level. The phylum, class, and family to which they belong are listed in the legend.

S2

Discussion:

LEfSe analysis revealed that 9 bacteria groups, including *Proteobacteria*, *Bacilli*, *Gammaproteobacteria,* and *Lactobacillales* increased in the CH group. Previous research indicates that *Proteobacteria* correlate with intestinal and hepatic inflammation, while *Lactobacillales* are linked to steatosis and fibrosis [1]. Furthermore, the study revealed that the enrichment of lactobacilli mediates the production of IL-17, contributing to the progression of liver fibrosis and inflammation [2]. Additionally, the CH group exhibited a marked reduction in 11 bacterial types, including *Bacteroidota*, *Clostridia*, *Oscillospirales*, *Lachnospirales*, *Ruminococcaceae,* and *Faecalibacterium*. *Ruminococcaceae* belongs to the phylum *Firmicutes*, and its reduction in the CH group indicates an exacerbation of inflammatory responses [3]. Research identifies a correlation between *Ruminococcaceae* and Th17 polarization, which promotes IL-17 production [4]. These findings indicate an intrinsic link among IL-17, gut microbiota, and CLD.

Reference

1 Korpela K, Mutanen A, Salonen A, Savilahti E, de Vos WM, Pakarinen MP. Intestinal Microbiota Signatures Associated With Histological Liver Steatosis in Pediatric-Onset Intestinal Failure. *J Parenter Enter Nutr* 2015; **41**: 238–248. [DOI: 10.1177/0148607115584388]

2 Tedesco D, Thapa M, Chin CY, Ge Y, Gong M, Li J, Gumber S, Speck P, Elrod EJ, Burd EM, Kitchens WH, Magliocca JF, Adams AB, Weiss DS, Mohamadzadeh M, Grakoui A. Alterations in Intestinal Microbiota Lead to Production of Interleukin 17 by Intrahepatic γδ T-Cell Receptor–Positive Cells and Pathogenesis of Cholestatic Liver Disease. *Gastroenterology* 2018; **154**: 2178–2193. [PMID: 29454797 DOI: 10.1053/j.gastro.2018.02.019]

3 Sinha SR, Haileselassie Y, Nguyen LP, Tropini C, Wang M, Becker LS, Sim D, Jarr K, Spear ET, Singh G, Namkoong H, Bittinger K, Fischbach MA, Sonnenburg JL, Habtezion A. Dysbiosis-Induced Secondary Bile Acid Deficiency Promotes Intestinal Inflammation. *Cell Host & Microbe* 2020; **27**: 659-670.e5. [DOI: 10.1016/j.chom.2020.01.021]

4 Robertson G, Leclercq I, Farrell GC. II. Cytochrome*P*-450 enzymes and oxidative stress. *Am J Physiol Liver Physiol* 2001; **281**: G1135–G1139. [DOI: 10.1152/ajpgi.2001.281.5.G1135]

S3: Accession numbers:

Library ID	Accession numbers
CK.1	SRR33577888
CK.2	SRR33577887
CK.3	SRR33577876
CK.4	SRR33577865
CK.5	SRR33577862
CK.6	SRR33577861
CK.7	SRR33577860
CK.8	SRR33577859
CK.9	SRR33577858
CK.10	SRR33577857
CK.11	SRR33577886
CH.1	SRR33577885
CH.2	SRR33577884
CH.3	SRR33577883
CH.4	SRR33577882
CH.5	SRR33577881
CH.6	SRR33577880
CH.7	SRR33577879
CH.8	SRR33577878
CH.9	SRR33577877
CH.10	SRR33577875
CH.11	SRR33577874
CH.12	SRR33577873
CH.13	SRR33577872
CH.14	SRR33577871
CH.15	SRR33577870
CH.16	SRR33577869
CH.17	SRR33577868
CH.18	SRR33577867
CH.19	SRR33577866
CH.20	SRR33577864
CH.21	SRR33577863
